# *CasaABEn:* memorial of the history of Brazilian nursing in metaverse technology

**DOI:** 10.1590/0034-7167.20267901

**Published:** 2026-01-09

**Authors:** Jacinta de Fatima Sena da Silva, Maria Itayra Padilha, Osvaldo Peralta Bonetti, Fernanda Batista Oliveira Santos, Fabiane Ferraz, Sonia Acioli

**Affiliations:** I Fiocruz School of Government, Professor of the Master’s Degree in Public Health Policies, Angicos and PSAT. Universidade de Brasília, Department of Collective Health, Researcher and Professor. President of the Brazilian Nursing Association (2022-2025 Term). Brasília, Federal District, Brazil; II Universidade Federal de Santa Catarina, Graduate Program in Nursing. Member of the Construction Team for the Memorial CasaABEn Project, Museum in the Metaverse. Florianópolis, Santa Catarina, Brazil; III Fiocruz-Brasília School of Government, Angicos Center. Brasília, Federal District, Brazil; IV Universidade Federal de Minas Gerais, School of Nursing. Member of the Executive Committee of the Nursing History Showcase of the Virtual Health Library and Coordinator of the Scientific Department of Nursing History of the Brazilian Nursing Association (2023-2026 Term). Belo Horizonte, Minas Gerais, Brazil; V Universidade do Extremo Sul Catarinense, Graduate Program in Public Health. Criciúma, Santa Catarina, Brazil.; V Universidade do Estado do Rio de Janeiro, Biomedical Center, Faculty of Nursing. Coordinator of the Scientific Department of Nursing in Primary Care at ABEn National - 2022-2025 Term. Rio de Janeiro, Rio de Janeiro, Brazil

Amidst the celebrations of the centenary of the Brazilian Nursing Association (In Portuguese, *Associação Brasileira de Enfermagem* - ABEn), the ABEn National 2023-2025 board of directors has been making efforts to publicize the entity’s past achievements and contributions to strengthening professional practices in the field, realizing the right to health, and defending a sovereign, just, and democratic country. Therefore, this celebration translates into a reaffirmation of commitments to building an increasingly just, dignified, and democratic future for nursing professionals and for all Brazilians.

There is so much to celebrate in ABEn’s 100-year history, which is a pioneering and matriarchal entity in Brazilian nursing’s technical, political, scientific, and cultural organization. It is important to mention that ABEn, as a representative of nursing, in addition to caring for the population, also carries in its history indelible contributions to the achievement of women’s leadership in our country, as it is an identifiably female profession.

During its century-long history, the organization changed its name four times before becoming known as ABEn in 1954. Its national headquarters were located in Rio de Janeiro and Brasília^(1)^. Nationally, it was led by 24 presidents who, along with their respective boards of directors, developed and established a history of struggles, teachings, and lessons on how to structure a professional field based on principles and knowledge that shape the scientific field of nursing, engaged with the reality and with the social, economic, and political transformations of the country. Throughout its existence, ABEn has established itself as a social practice that aligns with the real demands of the Brazilian population and is committed to inventing new, viable approaches to overcoming inequalities and inequities in health and well-being.

Over the years, ABEn’s role has expanded, gradually taking over as guardian of the history and defender of the quality of nursing education and professional practice in Brazil, participating in all major social and political movements in favor of nursing development and the right to health. Nursing’s history is a powerful instrument of power. When valued, preserved, and disseminated, it builds professional identity, confers prestige on the group, and underpins critical reflection on professional practice. This process enables us to envision a promising future based on the teachings of pioneers like our first president: Edith de Magalhães Fraenkel^(2).^

The visionary project to create an ABEn museum took its first steps in 2022, stemming from the desire to continue preserving history and building upon the Nursing Memory Center, which was inaugurated in 2010. From then on, the search for technical, scientific, and financial feasibility brought ABEn closer to the Fundação Oswaldo Cruz (Fiocruz) in Brasília through the Angicos^1^ nucleus, which presents the proposed parliamentary amendment project to then federal deputy and nurse Carmen Emília Bonfá Zanotto, with the realization of the project in a metaverse environment occurring through the support and partnership of the Universidade do Extremo Sul Catarinense (UNESC), whose rector was nurse Luciane Bisognin Ceretta.

The *CasaABEn* project’s main objective is to organize the documentary, iconographic, and instrumental collections, as well as the narratives of those who constituted ABEn, in a safe and appropriate manner, aiming to build an archive in metaverse technology that promotes Brazilian nursing history preservation, as well as accessible communication and dissemination of information sources to the community.

ABEn’s history, presented in the metaverse environment^(3)^, aims to highlight ABEn’s leading role and pioneering spirit in its interactions with the evolutionary movements of Brazilian nursing. These movements demonstrate ABEn’s inextricable link to nursing professionals, education, research, professional practices, political and social movements, union and student organization, and the Cofen/Corens regulatory and oversight system, contributing to the formation of professional identity processes and the organization of Brazilian nursing.

The Brazilian nursing history metaverse, called Memorial *CasaABEn*, presents itself as a source of consultation, research, and dissemination of knowledge for the community and for Brazilian and international nursing, which takes place through visits to the metaverse environment, which is accessible both with the use of virtual reality glasses and through download, enabling inclusion and the right of everyone to learn about this history through a wide variety of electronic devices.

After much collective reflection and discussion, the chosen name is offered based on the understanding that this historical ABEn building is collective heritage, a “common home for nursing and society”. The name alludes to the notable ABEn building in Brasília, which has recently been revitalized and is intertwined with the political, cultural, and architectural history of the capital city.

The metaverse is an immersive virtual environment that blends the real and virtual worlds, considered a technological evolution. It can be accessed through devices that allow users to experience virtual and augmented reality. It allows people to meet, talk, and even create shared content, regardless of their physical location.

The Memorial *CasaABEn* introduces itself to the public through thematic trails that address key aspects of ABEn’s importance to Brazilian nursing from its founding in 1926 to the present day. These trails are:

TRAIL 1 - History, memory, and symbolism of Brazilian nursing: on this trail, visitors will find the Gallery of Presidents, the national headquarters of ABEn, the medallion used by presidents when they take office, and the ABEn emblem;

TRAIL 2 - ABEn’s participation in the formulation of policies for the training and education of Brazilian nurses: a trail organized into four historical periods (1926-1949, 1950-1972, 1973-1990, and 1991-2024), which allow visitors to learn about the history of nursing education initiatives at all levels of training, spearheaded by ABEn throughout its existence, and how much the association has been and remains committed to the quality of nursing education in Brazil.

TRAIL 3 - Nursing’s leading role during the COVID-19 pandemic: demonstrates ABEn’s and Brazilian nursing’s exemplary and courageous participation during the greatest epidemic of the 21^st^ century, highlighting nursing’s essential and structural role and its representative entities, especially ABEn, as well as the workers themselves, in dialogue with social movements and health organizations during the political and health crisis, also contributing to the preservation of the memory of this challenging historical period for Brazilian public health and nursing, emphasizing the performance of nursing during the pandemic.

In this context, these trails engage in dialogue and contribute to ABEn’s efforts in health and education policy and healthcare and treatment practices, being contextualized in convergence with the democratic struggles experienced in the country. It is worth noting that the construction of the metaverse is perceived as a continuous process that will undergo adjustments and reformulations. Therefore, new steps must be systematized so that the *Memorial CasaABEn* space more faithfully expresses the richness of ABEn’s and Brazilian nursing’s history, encompassing the record of the past and the present, and committed to future achievements.

Building the trails required a team effort involving the term team of ABEn National and Fiocruz, nursing historians, consulting teams for the trails, support teams from ABEn, Fiocruz and UNESC, communication advisors from ABEn and Fiocruz, and professionals in journalism, communication, and metaverse technology developers.

In order to ensure fidelity to the facts and achievements and contribute to the systematization processes and recovery of possible gaps in our historical record, the *CasaABEn* project is being developed through a participatory process, inaugurating, in its first steps, a Board of Trustees in which all ABEn’s honorary presidents participate, in addition to the national board of directors for the 2023-2025 term.

To this end, weekly meetings were held between and with the teams to formulate the content to be produced by historians, which was then presented to the developers, who implemented it in the metaverse environment and made it available to the public. Documentary and iconographic sources were consulted, especially: documentaries about ABEn; more than 1,000 photos from the ABEn National and Section archives on the selected themes; researchers’ personal archives; more than 100 articles published about ABEn and about the biographies of the presidents; verification of the administrations with the presidents and their boards of directors; editorials and articles published in the Brazilian Journal of Nursing (In Portuguese, *Revista Brasileira de Enfermagem*); and minutes books of ABEn related to the Brazilian National Councils of the Brazilian Nursing Association, Brazilian National Assembly of Delegates, proceedings of Brazilian Nursing Congresses, Brazilian National Seminar on Nursing Research, Brazilian National Seminar on Guidelines for Nursing Education, among others. In addition, an extensive media survey was conducted, selecting 236 news articles published on the ABEn network and 801 news articles published by representative health and nursing organizations from 2020 to 2023 (the period of the pandemic). Furthermore, two workshops were held with the presidents and the Board of Trustees to align the development of the trails. Interviews were also conducted with the 11 ABEn presidents who continue to make history, namely Rosa Godoy, Sonia Acioli de Oliveira, Angela Maria Alvarez, Eucléa Gomes Vale, Maria Goretti David Lopes, Ivone Evangelista Cabral, Maria José Rossi, Maria Auxiliadora Christófaro, Ieda de Alencar Barreira, Stella Maria Fernandes de Barros, and Francisca Valda da Silva, who made it possible to record facts and impressions based on the words of those who built and continue to build ABEn’s history. In addition, in a second phase, directors of education from ABEn National, such as Elizabeth Teixeira and Edlamar Kátia Adami, were also interviewed.

*CasaABEn* understands that valuing the history of nursing and the collective memory of this Brazilian social heritage- ABEn - is paramount. Preservation spaces allow for critical perspectives on historical movements in professional development. They help foster reflective understanding of the present and facilitate connections and identification between professionals and society. Additionally, they support the development of possible and desirable alternatives for advancing nursing with social responsibility and valuing the wholeness of the human being who is cared for.

It is expected that the unique contact with ABEn’s and Brazilian nursing’s history, provided by the visit to the *Memorial CasaABEn*, promote and mobilize the senses beyond listening and reading, further strengthening interest, engagement, and fascination with the evolutionary steps, achievements, and transformations that have occurred in the last hundred years. May *CasaABEn* bring everyone closer together and instill in them a sense of belonging to ABEn and nursing practices, shaped by a political project of health, care, and a more just, supportive, ethical, and democratic society. May these visits serve as a beacon for constructing the desired future!
