# Supplementary material for: CasaABEn: memorial of the history of Brazilian nursing in metaverse technology
Source: Rev Bras Enferm. 2026 Jan 9;79:e7901. doi: 10.1590/0034-7167.20267901 (PMC12818049; doi:10.1590/0034-7167.20267901)
Supplement: Translation

# Translation: CasaABEn: memorial de la historia de la enfermería brasileña en la tecnología del metaverso

**DOI:** 10.1590/0034-7167.20267901es

**Authors:** Jacinta de Fatima Sena da Silva, Maria Itayra Padilha, Osvaldo Peralta Bonetti, Fernanda Batista Oliveira Santos, Fabiane Ferraz, Sonia Acioli

**Affiliations:** I Escuela de Gobierno Fiocruz-Brasília, Profesora, Maestría en Políticas de Salud Pública. Universidade de Brasília, Departamento de Salud Pública, investigador y profesor colaborador. Presidente de ABEn (2022-2025). Brasilia, Distrito Federal, Brasil.; II Universidade Federal de Santa Catarina, Programa de Postgrado en Enfermería. Miembro del Equipo de Construcción del Proyecto Memorial CASA ABEn, Museo en Metaverso. Florianópolis, Santa Catarina, Brasil; III Escuela de Gobierno Fiocruz-Brasília, Centro Angicos. Brasilia, Distrito Federal, Brasil; IV Universidade Federal de Minas Gerais, Facultad de Enfermería. Miembro del Comité Ejecutivo de la Muestra de Historia de la Enfermería de la Biblioteca Virtual de Salud y Coordinadora del Departamento Científico de Historia de la Enfermería de la Asociación Nacional de Enfermería de Brasil (Gestión 2023-2026). Belo Horizonte, Minas Gerais, Brasil; V Universidade do Extremo Sul Catarinense, Programa de Posgrado en Salud Pública. Criciúma, Santa Catarina, Brasil; VI Universidade do Estado do Rio de Janeiro, Centro Biomédico, Escuela de Enfermería. Coordinadora del Departamento Científico de Enfermería en Atención Primaria de la ABEn Nacional - Gestión 2022-2025. Rio de Janeiro, Rio de Janeiro, Brasil

En el marco de las conmemoraciones del centenario de la Asociación Brasileña de Enfermería (En portugués, *Associação Brasileira de Enfermagem* - ABEn), la dirección de la ABEn Nacional, gestión 2023-2025, ha realizado esfuerzos para divulgar las conquistas pasadas de la entidad y sus contribuciones al fortalecimiento del ejercicio profesional de la categoría, la realización del derecho a la salud y las luchas en defensa de un país soberano, justo y democrático. Por tanto, esta celebración se traduce en una reafirmación de los compromisos con la construcción de un futuro cada vez más justo, digno y democrático para los trabajadores de enfermería y para todos los brasileños.

Tenemos mucho que celebrar en los 100 años de historia de la ABEn, entidad pionera y matriz de la organización técnica, política, científica y cultural de la enfermería brasileña. Es importante mencionar que la ABEn, como representación de la enfermería, además de atender a la población, también aporta en su historia contribuciones indelebles al logro del protagonismo de las mujeres en nuestro país, al ser una categoría con identidad femenina.

Durante su centenaria trayectoria, la organización cambió de nombre cuatro veces hasta 1954, cuando se convirtió en ABEn. Tenía sus sedes nacionales en Rio de Janeiro y Brasília^(1)^. Fue liderada a nivel nacional por 24 presidentes, quienes, con sus respectivos directores, desarrollaron e imprimieron una historia de luchas, enseñanzas y aprendizajes sobre cómo estructurar un campo profesional con principios de valores y conocimientos que moldean el campo científico de la enfermería involucrado con la realidad y con las transformaciones sociales, económicas y políticas del país. Así, la ABEn, a lo largo de su existencia, se ha constituido como una práctica social alineada a las demandas reales de la población brasileña y comprometida con la invención de innovaciones viables para superar las desigualdades e inequidades en salud y bienestar.

Con el paso de los años, el rol de la ABEn se ha expandido, asumiendo gradualmente el rol de guardiana de la historia y defensora de la calidad de la educación y la práctica profesional de enfermería en Brasil, participando en los principales movimientos sociales y políticos que promueven el desarrollo de la enfermería y el derecho a la salud. La historia de la profesión es un poderoso instrumento de poder. Cuando se valora, preserva y difunde, no solo construye la identidad profesional y confiere prestigio al grupo, sino que también sustenta la reflexión crítica sobre la práctica profesional. Este proceso nos permite vislumbrar un futuro prometedor, siguiendo las enseñanzas de pioneras como nuestra primera presidenta: Edith de Magalhães Fraenkel^(2)^.

El proyecto visionario de crear un museo de la ABEn tiene sus primeros pasos delineados en 2022, basado en la sensibilidad de dar continuidad al trabajo de preservación de la historia del Centro de Memoria de Enfermería, inaugurado en 2010. A partir de entonces, la búsqueda de viabilidad técnica, científica y financiera acercó la ABEn a la *Fundação Oswaldo Cruz* (Fiocruz) Brasília a través del núcleo Angicos^1^, que presenta la propuesta de proyecto de enmienda parlamentaria a la entonces diputada enfermera federal Carmen Emília Bonfá Zanotto, y la materialización del proyecto en un entorno de metaverso ocurre gracias al apoyo y asociación de la *Universidade do Extremo Sul Catarinense* (UNESC), cuya rectora fue la enfermera Luciane Bisognin Ceretta.

El proyecto *CasaABEn* tiene como principal objetivo organizar, de forma segura y adecuada, los acervos documentales, iconográficos, instrumentales y las narrativas de quienes constituyeron la ABEn, buscando construir un acervo en tecnología de metaverso que promueva la preservación de la historia de la enfermería brasileña, así como la comunicación y difusión accesible de fuentes de información a la comunidad.

La historia de ABEn, presentada en el entorno del metaverso^(3)^, busca destacar el protagonismo y el espíritu pionero expresados en las interrelaciones de la ABEn con los movimientos evolutivos de la enfermería brasileña. Estos movimientos expresan la inseparabilidad de la ABEn en todo lo relacionado con los profesionales de enfermería, la educación, la investigación, las prácticas profesionales, los movimientos políticos, sociales y culturales, la estructuración de los movimientos sindicales y estudiantiles, y el sistema de inspección y normalización Cofen/Corens, contribuyendo a la formación de procesos de identidad profesional y a la organización del campo de la enfermería brasileña.

El metaverso de la historia de la enfermería brasileña, denominado *Memorial CasaABEn*, se presenta como una fuente de consulta, investigación y difusión de conocimiento para la comunidad y para la enfermería brasileña e internacional, que ocurre a través de una visita al entorno del metaverso, accesible tanto con el uso de gafas de realidad virtual como mediante descarga, posibilitando la inclusión y el derecho de todos a conocer esta historia a través de los más variados dispositivos electrónicos.

El nombre, concebido tras una intensa reflexión y debate colectivo, se basa en la comprensión de que este histórico edificio de la ABEn es un patrimonio colectivo, “un espacio compartido para la enfermería y la sociedad”. Su entorno evoca el memorable edificio de la ABEn en Brasília, recientemente revitalizado, que también se entrelaza con la historia política, cultural y arquitectónica de la capital de la república.

El metaverso es un entorno virtual inmersivo que fusiona el mundo real y el virtual, considerado un avance tecnológico. Se puede acceder a él mediante dispositivos que permiten experiencias de realidad virtual y aumentada. Permite a las personas reunirse, chatear e incluso crear contenido compartido, independientemente de su ubicación física.

El *Memorial CasaABEn* se presenta al público a través de recorridos que abordan aspectos clave para comprender la importancia de la ABEn para la enfermería brasileña desde su fundación en 1926 hasta la actualidad. Estos son:

RECORRIDO 1 - Historia, memoria y símbolos de la enfermería brasileña: en este recorrido, el visitante encontrará la Galería de Presidentes, la sede nacional de la ABEn, la medalla que usaron los presidentes al tomar posesión del cargo y el emblema de la ABEn;

RECORRIDO 2 - Participación de la ABEn en la formulación de políticas de formación y educación para la enfermería brasileña: un recorrido organizado en cuatro secciones históricas (1926-1949, 1950-1972, 1973-1990 y 1991-2024), que permiten al visitante conocer la historia de las acciones de educación en enfermería en todos los niveles de formación, lideradas por la ABEn a lo largo de su existencia, y cuánto la asociación se involucró y sigue comprometida con la calidad de la formación en enfermería en Brasil.

RECORRIDO 3 - El papel protagónico de la enfermería en la pandemia de COVID-19: demuestra la participación ejemplar y valiente de la ABEn y de la enfermería brasileña durante la mayor epidemia del siglo XXI, destacando el papel esencial y estructurante de la categoría, de sus entidades representativas, especialmente la ABEn, así como de los trabajadores, en diálogo con los movimientos sociales y entidades del campo de la salud durante la crisis política y sanitaria, contribuyendo también a la preservación de la memoria de este desafiante período histórico de la salud pública brasileña y de la profesión, destacando el papel de la enfermería durante la pandemia.

En este contexto, estos viajes discuten y demuestran la contribución de la ABEn a las políticas de salud y educación, así como a las prácticas de atención sanitaria, en consonancia con las luchas democráticas del país. Cabe destacar que la construcción del metaverso se percibe como un proceso continuo que experimentará ajustes y reformulaciones. Por eso, es necesario sistematizar nuevos pasos, para que el espacio *Memorial CasaABEn* exprese con mayor fidelidad la rica historia de la ABEn y de la enfermería brasileña, reunida en el registro del pasado y del presente, y comprometida con realizaciones futuras.

El proceso de construcción de los senderos requirió un trabajo en equipo que involucró al equipo gestor de la ABEn Nacional y de Fiocruz, historiadores de enfermería, equipos de consultoría de los senderos, equipos de apoyo de la ABEn, de Fiocruz y de la UNESC, asesores de comunicación de la ABEn y de Fiocruz y profesionales del periodismo, de la comunicación y desarrolladores de tecnología de metaverso.

Con el fin de asegurar la fidelidad a los hechos y logros, así como contribuir a los procesos de sistematización y recuperación de posibles vacíos en el registro de nuestra historia, el desarrollo del proyecto *CasaABEn* se materializa a través de la gestión participativa, inaugurando, en sus primeros pasos, un Patronato con la participación de todos los presidentes honorarios de la ABEn, además de la junta directiva nacional para la gestión 2023-2025.

Para ello, se realizaron reuniones semanales entre y con los equipos para formular el contenido a producir por los historiadores, que posteriormente fue presentado a los desarrolladores para que lo imprimieran en el entorno del metaverso y lo pusieran a disposición del público. Se consultaron fuentes documentales e iconográficas, especialmente: documentales sobre la ABEn; más de 1.000 fotografías de la colección ABEn Nacional y Secciones sobre temas seleccionados; colecciones personales de investigadores; más de 100 artículos publicados sobre ABEn y biografías de los presidentes; verificación de la gestión con los presidentes y sus juntas directivas; Editoriales y artículos publicados en la Revista Brasileña de Enfermería (En portugués, *Revista Brasileira de Enfermagem*); y libros de actas de la ABEn relacionados con los Consejos Nacionales de la Asociación Brasileña de Enfermería, Asamblea Nacional de Delegados, actas de los Congresos Brasileños de Enfermería, Seminario Nacional de Investigación en Enfermería, Seminario Nacional de Directrices para la Educación en Enfermería, entre otros. Además, se realizó un amplio relevamiento de medios, seleccionándose 236 noticias publicadas en la red ABEn y 801 noticias publicadas en entidades representativas de la salud y enfermería de 2020 a 2023 (periodo de pandemia). Además, se realizaron dos talleres con los presidentes y Patronato para alinear el desarrollo de los senderos, y también se realizaron entrevistas a los 11 presidentes de la ABEn que siguen haciendo historia, a saber, Rosa Godoy, Sonia Acioli de Oliveira, Angela Maria Alvarez, Eucléa Gomes Vale, Maria Goretti David Lopes, Ivone Evangelista Cabral, Maria José Rossi, Maria Auxiliadora Christófaro, Ieda de Alencar Barreira, Stella María Fernandes de Barros y Francisca Valda da Silva, quienes permitieron registrar hechos e impresiones basados en las palabras de quienes construyeron y siguen construyendo la historia de ABEn. Además, en un segundo momento, también se entrevistó a directores de educación de ABEn Nacional, como Elizabeth Teixeira y Edlamar Kátia Adami.

*CasaABEn* expresa la comprensión de que valorar la historia de la enfermería y la memoria colectiva de este patrimonio social brasileño -ABEn- es fundamental. Los espacios de preservación permiten una mirada crítica a los movimientos históricos del desarrollo profesional, ayudando a comprender el presente de forma reflexiva, fomentando las conexiones y la identificación entre los profesionales y la sociedad, y apoyando el desarrollo de alternativas posibles y deseables para el avance de la enfermería con responsabilidad social, valorando la integralidad del ser humano que recibe los cuidados.

Se espera que el contacto diferenciado con la historia de la ABEn y de la enfermería brasileña, proporcionado por la visita al *Memorial CasaABEn*, promueva y mueva más los sentidos de escucha y lectura, fortaleciendo aún más el interés, el compromiso y el encantamiento con los pasos evolutivos, así como con las conquistas y transformaciones realizadas en este siglo de historias. Que *CasaABEn* acerque a todos e infunda un sentido de pertenencia a la práctica y la profesión de ABEn, forjadas por un proyecto político de salud, atención y una sociedad más justa, solidaria, ética y democrática. ¡Que las visitas sirvan como faro para construir el futuro deseado!

# Translation: CasaABEn: memorial da história da enfermagem brasileira na tecnologia metaverso

**DOI:** 10.1590/0034-7167.20267901pt

**Authors:** Jacinta de Fatima Sena da Silva, Maria Itayra Padilha, Osvaldo Peralta Bonetti, Fernanda Batista Oliveira Santos, Fabiane Ferraz, Sonia Acioli

**Affiliations:** I Escola de Governo da Fiocruz-Brasília, Docente Mestrado em Políticas Públicas de Saúde, Angicos e PSAT. Universidade de Brasília, Departamento de Saúde Coletiva, pesquisadora e docente colaboradora. Presidenta da ABEn (Gestão 2022-2025). Brasília, Distrito Federal, Brasil; II Universidade Federal de Santa Catarina, Programa de Pós-graduação em Enfermagem. Membro da Equipe de Construção do Projeto Memorial CASA ABEn, Museu em Metaverso. Florianópolis, Santa Catarina, Brasil; III Escola de Governo da Fiocruz-Brasília, Núcleo Angicos. Brasília, Distrito Federal, Brasil; IV Universidade Federal de Minas Gerais, Escola de Enfermagem. Membro da Comissão Executiva da Vitrine de História da Enfermagem da Biblioteca Virtual de Saúde e Coordenadora do Departamento Científico de História da Enfermagem da Associação Brasileira de Enfermagem Nacional (Gestão 2023-2026). Belo Horizonte, Minas Gerais, Brasil; V Universidade do Extremo Sul Catarinense, Programa de Pós-Graduação em Saúde Coletiva. Criciúma, Santa Catarina, Brasil; VI Universidade do Estado do Rio de Janeiro, Centro Biomédico, Faculdade de Enfermagem. Coordenadora do Departamento Científico de Enfermagem na Atenção Básica da ABEn Nacional - Gestão 2022-2025. Rio de Janeiro, Rio de Janeiro, Brasil

No enlevo das celebrações do centenário da Associação Brasileira de Enfermagem (ABEn), a diretoria da ABEn Nacional, gestão 2023-2025, tem envidado esforços para publicizar o passado de conquistas e contribuições da entidade para o fortalecimento da prática profissional da categoria, da efetivação do direito à saúde e das lutas em defesa de um país soberano, justo e democrático. Logo, tal celebração se traduz na reafirmação de compromissos com a construção de um futuro cada vez mais justo, digno e democrático para os trabalhadores da enfermagem e para todos os brasileiros.

Temos muito a celebrar na trajetória destes 100 anos da ABEn, entidade pioneira e matriarca da organização técnica, política, científica e cultural da enfermagem brasileira. Importa mencionar que a ABEn, enquanto representação da enfermagem, além do cuidado com a população, também carrega em seu histórico contribuições indeléveis com a conquista do protagonismo das mulheres em nosso país, por ser uma categoria identitariamente feminina.

Em seu percurso centenário, a entidade mudou quatro vezes de nome até 1954, quando passou a ser denominada ABEn. Teve suas sedes nacionais no Rio de Janeiro e em Brasília^(1)^. Foi conduzida nacionalmente por 24 presidentas, as quais, com suas respectivas diretorias, desenvolveram e imprimiram um histórico de lutas, ensinamentos e aprendizados sobre como estruturar um campo profissional com princípios valorativos e conhecimentos que conformam o campo científico da enfermagem implicada com a realidade e com as transformações sociais, econômicas e políticas do país. Assim, a ABEn, ao longo de sua existência, constitui-se como uma prática social alinhada com as reais demandas da população brasileira e comprometida com a invenção de inéditos viáveis para superação das desigualdades e das iniquidades em saúde e bem viver.

Com o passar dos anos, tivemos uma ampliação do papel da ABEn, que gradualmente assumiu o papel de guardiã da história e defensora da qualidade do ensino e da prática profissional da enfermagem no Brasil, participando de todos os grandes movimentos sociais e políticos em prol do desenvolvimento da enfermagem e do direito à saúde. A história da profissão é um poderoso instrumento de poder. Ao ser valorizada, preservada e divulgada, ela não apenas constrói a identidade profissional e confere prestígio ao grupo, mas também alicerça a reflexão crítica sobre a prática profissional. Este processo permite vislumbrar um futuro promissor, seguindo os ensinamentos deixados por precursoras como nossa primeira presidenta: Edith de Magalhães Fraenkel^(2)^.

O projeto visionário de criar um museu da ABEn tem seus primeiros passos esboçados ainda em 2022, a partir da sensibilidade de dar continuidade ao trabalho de preservação da história, do Centro de Memória da Enfermagem, inaugurado em 2010. A partir de então, a busca da viabilidade técnica, científica e financeira aproximou a ABEn da Fundação Oswaldo Cruz (Fiocruz) Brasília, por meio do núcleo Angicos^1^, que apresenta a proposta de projeto de emenda parlamentar à então deputada federal enfermeira Carmen Emília Bonfá Zanotto, sendo que a materialização do projeto em um ambiente metaverso ocorre por meio do apoio e parceria da Universidade do Extremo Sul Catarinense (UNESC), cuja reitora era a enfermeira Luciane Bisognin Ceretta.

O projeto CasaABEn apresenta como objetivo principal organizar, de forma segura e adequada, as coleções documentais, iconográficas, instrumentais e as narrativas daqueles que constituíram a ABEn, visando construir um acervo na tecnologia metaverso que promova a preservação da história da enfermagem brasileira, bem como a comunicação e difusão acessíveis das fontes de informação à comunidade.

A história da ABEn, apresentada no ambiente metaverso^(3)^, busca visibilizar o protagonismo e o pioneirismo expressos nas interfaces da ABEn com os movimentos evolutivos pelos quais passa a enfermagem brasileira. Tais movimentos expressam uma indissociabilidade da ABEn em tudo o que se refere aos trabalhadores de enfermagem, à educação, à pesquisa, às práticas profissionais, aos movimentos políticos, sociais e culturais, à estruturação do movimento sindical, estudantil, e ao sistema de fiscalização e normatização Cofen/Corens, contribuindo para formação dos processos identitários profissionais e da organização do campo da enfermagem brasileira.

O metaverso da história da enfermagem brasileira, denominado Memorial CasaABEn, apresenta-se como uma fonte de consulta, pesquisa e difusão de conhecimentos para a comunidade e para a enfermagem brasileira e internacional, que ocorre por meio da visita ao ambiente metaverso, o qual é acessível tanto com o uso de óculos de realidade virtual quanto por meio de *download*, possibilitando a inclusão e direito de todos a conhecer esta história pelos mais variados dispositivos eletrônicos.

O nome idealizado, depois de muitas reflexões e problematizações coletivas, é ofertado a partir da compreensão de que esta construção histórica da ABEn é patrimônio coletivo, sendo “uma casa comum da enfermagem e da sociedade”. Já sua ambientação alude ao memorável prédio da ABEn em Brasília, recentemente revitalizado, o qual se confunde também com a própria história política, cultural e arquitetônica da capital da república.

O metaverso é um ambiente virtual imersivo que mistura o mundo real e o virtual, considerado uma evolução tecnológica. Pode ser acessado por meio de dispositivos que permitam vivenciar a realidade virtual e aumentada. Permite que as pessoas se encontrem, conversem e até mesmo criem conteúdos compartilhados, independentemente de onde estejam localizadas fisicamente.

O Memorial CasaABEn se apresenta ao público por meio de trilhas, abordando aspectos centrais para a compreensão da importância da ABEn para a enfermagem brasileira desde a sua fundação, em 1926, até os dias atuais. São elas:

TRILHA 1 - História, memória e simbologias da enfermagem brasileira: nesta trilha, o visitante irá encontrar a Galeria das Presidentas, as sedes nacionais da ABEn, o medalhão utilizado pelas presidentas ao serem empossadas e o emblema da ABEn;

TRILHA 2 - Participação da ABEn na formulação de políticas de formação e educação da enfermagem brasileira: trilha organizada em quatro recortes históricos (1926-1949, 1950-1972, 1973-1990 e 1991-2024), os quais possibilitam o visitante conhecer a história das ações de educação em enfermagem em todos os níveis de formação, protagonizadas pela ABEn ao longo de sua existência, e o quanto a associação se implicou e segue comprometida com a qualidade da formação em enfermagem no Brasil.

TRILHA 3 - O protagonismo da enfermagem na pandemia de COVID-19: demonstra a participação exemplar e corajosa da ABEn e da enfermagem brasileira durante a maior epidemia do século XXI, ressaltando o papel essencial e estruturante da categoria, suas entidades representativas, em especial a ABEn, assim como dos trabalhadores, em diálogo com movimentos sociais e entidades do campo da saúde durante a crise política e sanitária, contribuindo também com a preservação da memória sobre esse desafiador período histórico da saúde pública brasileira e da profissão, ressaltando a atuação da enfermagem durante a pandemia.

Nesse contexto, essas trilhas dialogam e aportam a contribuição da ABEn às políticas de saúde e de educação, e às práticas de atenção e cuidado à saúde, sendo contextualizadas em convergência com as lutas democráticas vivenciadas no país. Merece destaque o fato de que a construção do metaverso é percebida como um processo contínuo que deverá sofrer adequações e reformulações. Logo, novos passos devem ser sistematizados, a fim de que o espaço do Memorial CasaABEn expresse com maior fidelidade a riqueza da história da ABEn e da enfermagem brasileira, congregada no registro do passado e do presente, e comprometida com conquistas do futuro.

O processo de construção das trilhas demandou um trabalho em equipe no qual participaram a equipe de gestão da ABEn Nacional e da Fiocruz, historiadores de enfermagem, equipes de consultoria para as trilhas, equipes de apoio da ABEn, Fiocruz e UNESC, assessores de comunicação da ABEn e da Fiocruz, e profissionais de jornalismo, comunicação e desenvolvedores da tecnologia do metaverso.

A fim de zelar pela fidelidade aos fatos e realizações, como também contribuir com os processos de sistematização e resgate de possíveis lacunas no registro de nossa história, o desenvolvimento do projeto CasaABEn é materializado por meio de uma gestão participativa, inaugurando, em seus primeiros passos, um Conselho Curador junto do qual participam todas as presidentas de honra da ABEn, além da diretoria nacional da gestão 2023-2025.

Para tanto, foram realizadas reuniões semanais entre e com as equipes para formular os conteúdos a serem produzidos pelas historiadoras, os quais posteriormente foram apresentados aos desenvolvedores para que os imprimissem no ambiente metaverso e estivessem disponibilizados ao público. Foram consultadas fontes documentais e iconográficas, especialmente: os documentários sobre a ABEn; mais de 1.000 fotos do acervo da ABEn Nacional e Seções sobre os temas selecionados; acervos pessoais das pesquisadoras; mais de 100 artigos publicados sobre a ABEn e sobre biografias das presidentas; checagem das gestões com as presidentas e suas diretorias; editoriais e artigos publicados na Revista Brasileira de Enfermagem; e livros de atas da ABEn relativos aos Conselhos Nacionais da Associação Brasileira de Enfermagem, Assembléia Nacional de Delegados, anais dos Congressos Brasileiros de Enfermagem, Seminário Nacional de Pesquisa em Enfermagem, Seminário Nacional de Diretrizes para a Educação em Enfermagem, entre outros. Além disso, foi realizado amplo levantamento midiático, tendo sido selecionadas 236 notícias publicadas na rede ABEn e 801 notícias publicadas em entidades representativas da saúde e da enfermagem no período de 2020 a 2023 (período da pandemia). Ainda, foram realizadas duas oficinas de trabalho com as presidentas e Conselho Curador para alinhamento do desenvolvimento das trilhas, e também realização de entrevistas com as 11 presidentas da ABEn que seguem fazendo história, sendo elas Rosa Godoy, Sonia Acioli de Oliveira, Angela Maria Alvarez, Eucléa Gomes Vale, Maria Goretti David Lopes, Ivone Evangelista Cabral, Maria José Rossi, Maria Auxiliadora Christófaro, Ieda de Alencar Barreira, Stella Maria Fernandes de Barros e Francisca Valda da Silva, que possibilitaram o registro de fatos e impressões a partir da fala daquelas que construíram e constroem a história da ABEn. Além destas, em um segundo momento, também foram entrevistadas diretoras de educação da ABEn Nacional, como Elizabeth Teixeira e Edlamar Kátia Adami.

A CasaABEn expressa a compreensão de que é primordial a valorização da história da enfermagem e da memória coletiva deste patrimônio social brasileiro - a ABEn. Espaços de preservação possibilitam olhar crítico acerca dos movimentos históricos da construção profissional, ajudando no entendimento do presente, de modo reflexivo, na vinculação e na identificação dos profissionais e da sociedade, além de apoiar a elaboração de alternativas possíveis e desejáveis para o avanço da enfermagem com responsabilidade social, valorizando a integralidade do ser humano que é cuidado.

Espera-se que o contato diferenciado com a história da ABEn e da enfermagem brasileira, proporcionado pela visitação ao Memorial CasaABEn, promova e mobilize sentidos para além da escuta e da leitura, fortalecendo ainda mais o interesse, engajamento e encantamento com os passos evolutivos, bem como as conquistas e transformações realizadas neste centenário de histórias. Que a CasaABEn possa aproximar e instigar em todos o sentido de pertencimento à práxis da ABEn e da categoria, conformadas em projeto político de saúde, cuidado e uma sociedade mais justa, solidária, ética e democrática. Que as visitações sirvam de farol à construção do desejado futuro!
